# Edge‐based effective active appearance model for real‐time wrinkle detection

**DOI:** 10.1111/srt.12977

**Published:** 2020-10-27

**Authors:** Umirzakova Sabina, Taeg Keun Whangbo

**Affiliations:** ^1^ Department of IT Convergence Engineering Gachon University Seongnam South Korea; ^2^ Department of Computer Science Gachon University Seongnam South Korea

**Keywords:** active appearance model, automatic wrinkle detection, face feature points, face landmark detection, facial wrinkles, nasolabial wrinkle line

## Abstract

**Background:**

Recently, the field of face and facial features has been progressively studied. The features of facial expression have gained increasing attention for related applications. The wrinkle is the most representative feature, and its research and applications have been topics of high interest. Wrinkles play an important role in face feature analysis. They have been widely used in applications, such as age estimation, skin texture classification, expression recognition, and simulation.

**Purpose:**

Existing approaches to the image‐based analysis of wrinkles as texture not as curvilinear discontinuity and wrinkle detection mainly have focused on detecting wrinkles on forehead position, which is usually horizontal linear shapes, while the detection of the nasolabial wrinkle is not well understood due to their variety of shapes and complexity.

**Method:**

In this paper, we present a nasolabial wrinkle line detecting effective algorithm based on the Active appearance model and Hessian filter to improve localization results by creating unique initial shapes of the wrinkle lines for each input face image.

**Results:**

Experimental results show that the proposed method is capable of tracking curve wrinkle lines, thus allowing to detect complexly structured wrinkle lines. This work demonstrates results illustrated the competitiveness of the proposed method in detecting nasolabial wrinkle lines.

**Conclusion:**

In our study, this was introduced the effectiveness of changing the structure of AAM and successfully applied in wrinkle line localizing, although competitive results are achieved by the proposed wrinkle detection method.

## 1 INTRODUCTION

The detection of wrinkle positions is critical for applications like facial beauty within which the positions of wrinkle lines and regions must be detected before they are removed or trimmed.^1^, ^2^ The quantitative evaluation of skin condition has been an area of quite an intense study. There is great interest in complementing the dermatologist's diagnostic visual assessment of skin with objective measures. These methods are also valuable for the efficient development of effective treatments.^3^ Facial skin wrinkles are not only important features in terms of facial aging and beauty but also can also provide cues to a person's lifestyle and health condition. For example, facial wrinkles can recognize facial expression,^4^, ^5^ or whether the person has been a smoker.^6^, ^7^ Some of the factors influencing facial wrinkles are a person's lifestyle, genetic inheritance, ethnicity, overall health, skincare routines, and gender. Hence, computer‐based analysis of facial wrinkles has great potential to exploit this underlying information for relevant applications. However, wrinkle regions continue to be manually located in various works, which could largely restrict their applicability. These wrinkle detection methods are limited to some simple wrinkles, and they are not enough accurate or general. To improve the efficiency of these methods, wrinkle detection should be automatic and general.

However, each method has its own strengths and weaknesses. Current wrinkle detection methods focused on detecting active wrinkle positions as forehead wrinkles,^8^, ^9^, ^10^ but detecting passive wrinkle position as cheek wrinkles is not effective with previous methods. The cheek wrinkles are more complicated and challenging. The above algorithms rely on a distinct boundary texture of the wrinkle region, and the information of texture around the wrinkle area is not sufficiently complete or effective for curve type wrinkle line detection.

To make nasolabial wrinkle detection method more efficient, this work is divided into two main parts:

1. Detecting initial position of wrinkle line by Hessian filter: To make wrinkle detection method more effective and common was detected candidate wrinkle position by applying a ridge detection algorithm.
2. Apply initial wrinkle line to Active Appearance Model as individual mean shape: Create an active shape model and localize the wrinkle line using initial shape of wrinkle line.

The main contribution of this work is summarized as follows: The information about related works on the wrinkle area was introduced in section 2. The proposed wrinkle detection method demonstrated in Section 3. Then, the experimental results and the corresponding figures are presented in Section 4. Finally, discussion and conclusion are in Section 5.

## 2 RELATED WORK

In this section, we introduce related work based on wrinkle line detection. We divided the section into three parts rely on methods that were used in the wrinkle research area: texture based, filters based, and shape model‐based.

### 2.1 Texture based

The automated location of wrinkles from images is an important step in age estimation. Ng et al^11^ studied a different wrinkle region extractor for age estimation. This method works by applied a Canny operator to detect the wrinkles and represented it as a pattern of age estimation. The edge detector detects the boundaries of the pattern, and it could not be suitable for wrinkle localization. Batool and Chellappa^12^ proposed to detect wrinkles by marked point processes, and the Markov chain Monte Carlo method was used to detect the initial positions of wrinkles. In Ref.,^1^ the authors proposed a method to detect forehead wrinkles, using a curve pattern as a soft biometric. The permanent wrinkles are often relatively simple with distinct shapes. Detection methods aiming at this type of wrinkle might not be useful for temporary wrinkles with nonlinear and blurry shapes.

### 2.2 Filters based

Localization wrinkle is a line or ridge detection problem. Only a few methods have been proposed in the previous. Ng et al^9^ introduced the detection of permanent wrinkles with a linear shape used a hybrid Hessian filter to locate the whole wrinkle line rather than the wrinkle edges. Forehead wrinkles were detected by growing and stitching wrinkle centerline parts extracted from filtered images of the maximum Gabor imitation with different thresholds.^13^ Cula et al^14^ proposed automatic detection of facial wrinkles, based on estimating the orientation and the frequency of elongated spatial features, captured via Gabor filtering of image. However, the above approaches are not efficient for blurry transient wrinkles, whose boundary edges might not be detected correctly by the edge detectors or filters.

### 2.3 Shape model‐based

Facial shape detection has received a lot of attention over the past decade and successfully applied in many research areas and have been topics of high interest using shape models to detect wrinkle line. The Active Shape Model (ASM) was used by training and locating 81 face feature points, including several points in the nasolabial region.^15^ In addition, wrinkles in some previously extracted fixed areas were detected using geometric elements such as a change in mean curvature. Wrinkle measurements are subsequently obtained using image gradient and surface curvature descriptors. Huang et al^16^ to detect wrinkles used active wavelet network with located feature points on relatively fixed positions and the deformable template model by replacing PCA‐based texture model with wavelet networking.

Current algorithms leave many opportunities to improve wrinkle detection method and introduced by the following drawbacks:

- Current wrinkle detectors rely on a bold boundary pattern of the wrinkle line.
- Texture and geometrical information around the wrinkle line not used completely.
- Above methods mainly suitable for detecting forehead wrinkle lines, wherein nasolabial wrinkle lines remain unexplored.

## 3 PROPOSED METHOD

**FIGURE 1:**
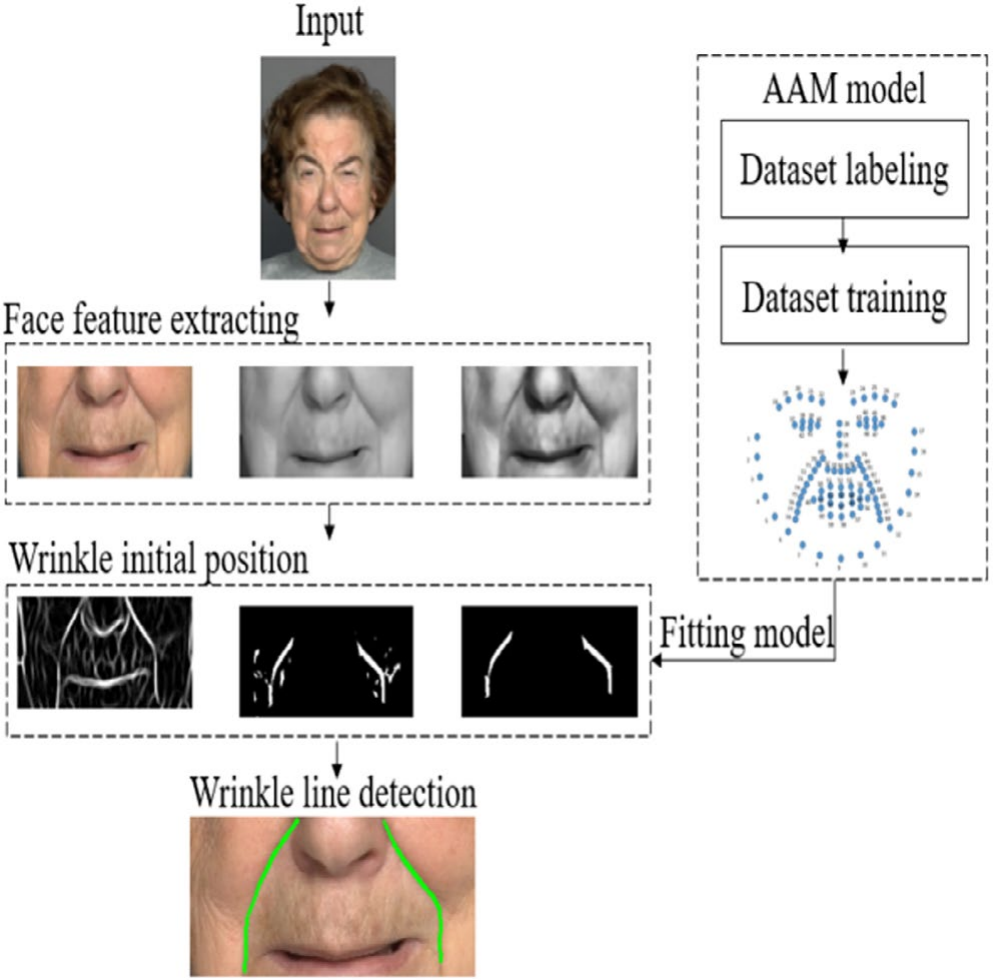
The flowchart of the proposed algorithm [Colour figure can be viewed at wileyonlinelibrary.com]

The proposed detector Figure 1 largely consists of three steps: first, detecting wrinkle position area; second, the ridge detection method is applied; and third, creating a unique initial shape and finally apply AAM using created initial shape.

### 3.1 Wrinkle edge detection

The first and most important step is to detect face and face feature points from the input image. In this step, using general AAM localized detect face and face features. The shape of the face represented as a sequence of 88 points (Figure 4), in which 68 points are face features, and 20 points are wrinkle line.

Chin region is initially extracted using points coordinates in Table 1. The extracted area covers the cheek, mouth, and nose regions, to remain only cheek part of the face, other regions overlay with the non‐zero mask, which allows concentrating only to the cheek region. Because human skin has many noises that impede detecting wrinkle line correctly, the Gaussian filter^17^ with histogram equalization^18^ is employed to balance skin noise.

**Table 1:** The borders of cheek area

Region	Points	Covering area
Cheek	2,5,11,14	Cheek, nose, and mouth
Left cheek	1:7,31,32,50,49	
Right cheek	15:11,35,53,54,64	

To extract the initial position of wrinkle lines in cheek area using edge detection operator is not effective, as edge detection operators detect borders between areas of high and low gray values, it could not detect wrinkle position. An edge detector is a first derivative operator, an edge detector measures the steepness of the slope at each point of the landscape. Our aim is thin lines are darker or brighter than their neighbors, this can be reached using second‐order derivatives. To localize wrinkles pixels by second‐order derivatives, we applied a Hessian filter. The Hessian filter is a matrix of second‐order derivative and is capable of capturing local structures in images. The eigenvalues of the Hessian filter evaluated at each image point quantify the rate of change of the gradient field in various directions. A small eigenvalue indicates a low rate of change in the field in the corresponding Eigen direction. The Hessian matrix *H* of the input image *I*, if image function is g in this case for small values *x* and *y* consisting of second‐order derivatives at scale *σ*, is given as:

(3.1) $$g\left(x,y\right)=\frac{1}{2}\left(\frac{x}{y}\right)*\left(\begin{array}{cc} \frac{\delta^{2}g}{\delta x^{2}} & \frac{\delta^{2}g}{\delta x\delta y}\\ \frac{\delta^{2}g}{\delta x\delta y} & \frac{\delta^{2}g}{\delta y^{2}} \end{array}\right)*\left(x,y\right)+\left(\frac{x}{y}\right)*\left(\frac{\delta g}{\delta x}\frac{\delta g}{\delta y}\right)+g\left(0,0\right)$$

(3.2) $$\left(\begin{array}{cc} \frac{\delta^{2}g}{\delta x^{2}} & \frac{\delta^{2}g}{\delta x\delta y}\\ \frac{\delta^{2}g}{\delta x\delta y} & \frac{\delta^{2}g}{\delta y^{2}} \end{array}\right)$$

In order to extract the Eigen direction in which a local structure of the image is decomposed, Equation (3.2) is transformed into the sum of eigenvalues *λ_1_, λ_2_* of the Hessian matrix:

(3.3) $$\lambda_{1}x^{2}+\lambda_{2}y^{2}$$

Using these values for areas, where the major eigenvalue of the Hessian is large. To localize the wrinkle line positions, we calculate the major eigenvalue at each pixel:

(3.4) $$\text{Value}=\text{Last}\left[\text{Eagenvalues}\left[\left(\begin{array}{cc} H_{\mathrm{xx}} & H_{\mathrm{xy}}\\ H_{\mathrm{xy}} & H_{\mathrm{yy}} \end{array}\right)\right]\right]$$

Then:

(3.5) $$\frac{1}{2}\left(H_{\mathrm{xx}}+H_{\mathrm{yy}}+\sqrt{H_{\mathrm{xx}}^{2}+4H_{\mathrm{xy}}^{2}+2H_{\mathrm{xx}}H_{\mathrm{yy}}+H_{\mathrm{yy}}^{2}}\right)$$

where *H_xx_, H_xy_, and H_yy_* are the second derivative.

### 3.2 Active appearance model

The wrinkle line structure of different persons might appear different and the wrinkle line might be weak or strong, as the cheek region is a passive part of the face in some cases just using second‐order derivatives is not enough to detect the whole line position. Detecting wrinkle with filters leads to losing some weak parts of the whole line, and an active appearance model (AAM) is applied to prevent such outcome and to find all of the candidate wrinkle lines. Active Appearance Model (AAM) introduced by Cootes et al.^19^ AAM is a statistics‐based pattern matching method in which shape and texture variability are extracted from a representative training set. Principal component analysis (PCA) according to the shape and texture data allows to get a parameterized face model that fully describes both trained faces and invisible with photorealistic quality. Fitting the AAM model to the target face is a nonlinear optimization task, where the difference in texture between the current model estimate and the target image covered by the model is minimized. AAM studying the correlations between texture residues and model parameters allows you to build a fast and efficient algorithm of steepest descent (SD) based on a fixed Jacobi matrix. During this process, model parameters were periodically reevaluated to better describe the target.

AAM was trained using the FACES dataset^20^ to face images with different emotional expressions. For detecting nasolabial wrinkles more accurately, have used local area features of the face, that labeled with a total of 88 points Figure 3, which 68 is present main face features (eyes, nose, mouth, jawline) and 20 points located in a wrinkle line. All labeled points represented as nk vector.

(3.6) $$x={\left(x_{1},y_{1},x_{2},y_{2},\ldots ,x_{n‐1},y_{n‐1},\right)}^{2}$$

The vector of labeled points Equation (3.6) used to create the mean shape of the trained dataset by Procrustes:

(3.7) $$\bar{x}=\frac{1}{n}\sum_{i=1}^{n}x_{i}$$

Procrustes transformation is applied to remove the difference between labeled shapes by the transformation of scaling translation and rotation Equation (3.7). Then, principal component analysis (PCA) is employed to keep the information about the identity of the pose and expression:

(3.8) $$S=\frac{1}{n‐1}\sum_{i=1}^{n}\left(x_{i}‐\bar{x}\right){\left(x_{i}‐\bar{x}\right)}^{T}$$

where S represents the eigenvectors of the shape, and $\bar{x}$ is the mean shape of Equation (3.8). By calculating this calculation, we take the mean shape of the database.

The quality of the AAM model fitting is very dependent of the initial estimate location:

Algorithm 1. AAM Iteration.

**Table:** 

*Iteration = 1* ***while*** *Iteration < MaxIteration or no improvement is made to error E_0_* ***do*** *Sample image at (x,y)*→*g_image_* *Build an AAM instance AAM (p)*→*(x_model_, y_model_, g_model_)* *Compute residual* $\partial g=g_{\mathrm{image}}‐g_{\mathrm{model}}$ *Evaluate Error* $E_{0}={\left|\partial g\right|}^{2}={\partial g\partial g}^{T}$ *Predict model displacements* $\partial p={\left(J^{T}J\right)}^{‐1}J^{T}\partial g$ *Set* α=1 *Update model parameters* $p_{k}=p_{k‐1}‐\alpha \partial p$ *Update sample control points from (x_model_, y_model_) with similarity* *compositional pose correction*→*(x_k_, y_k_)* *Sample image at (x_k_, y_k_)*→$g_{\mathrm{imagek}}$ *Compute residual* $\partial g_{k}=g_{\mathrm{imagek}}‐g_{\mathrm{model}}$ *Evaluate Error* $E_{k}=\partial g_{k}\partial g_{k}^{T}$ ***if*** *E_k_ < E_0_* ***then*** *Accept model parameters,* $p_{k}$ *Accept control points (x,y)* = *(x_k_, y_k_)* *Update current error E_k_ = E_0_* ***else*** *Try* α=1,α=0.5,α=0.25,α=0.125 ***end if*** *Iteration = Iteration +1* ***end while***

### 3.3 Fitting active appearance model

The AAM requires an initial estimate to the location of the shape, the better is this estimate, and minor is a risk of being trap in a local minimum. The disadvantage of AAM using for every input image only one created an initial mean shape of the trained database that brings failings in detecting various shape position of wrinkle line. Thus, Hessian filter was applied to create a unique wrinkle initial line for each input image, by wrinkle structures constructed to utilize the local deformation for shape variance modeling Table 1. Based on the detected edge, wrinkle lines using second‐order derivatives creating mean shape consist following steps:

Extract all detected wrinkle lines.

Discard some short and distorted lines less than *Line Length Threshold* (*LLT*) *LLT = 100*.

Remain wrinkle line with *Line Angle Threshold* (*LAT*) *less than LAT = π*.

(3.10) $$msh=\left\{\begin{array}{c} if\;extracted\;edges<LLT\\ if\;edge\;angel<LAT \end{array}\right)$$

**FIGURE 2:**
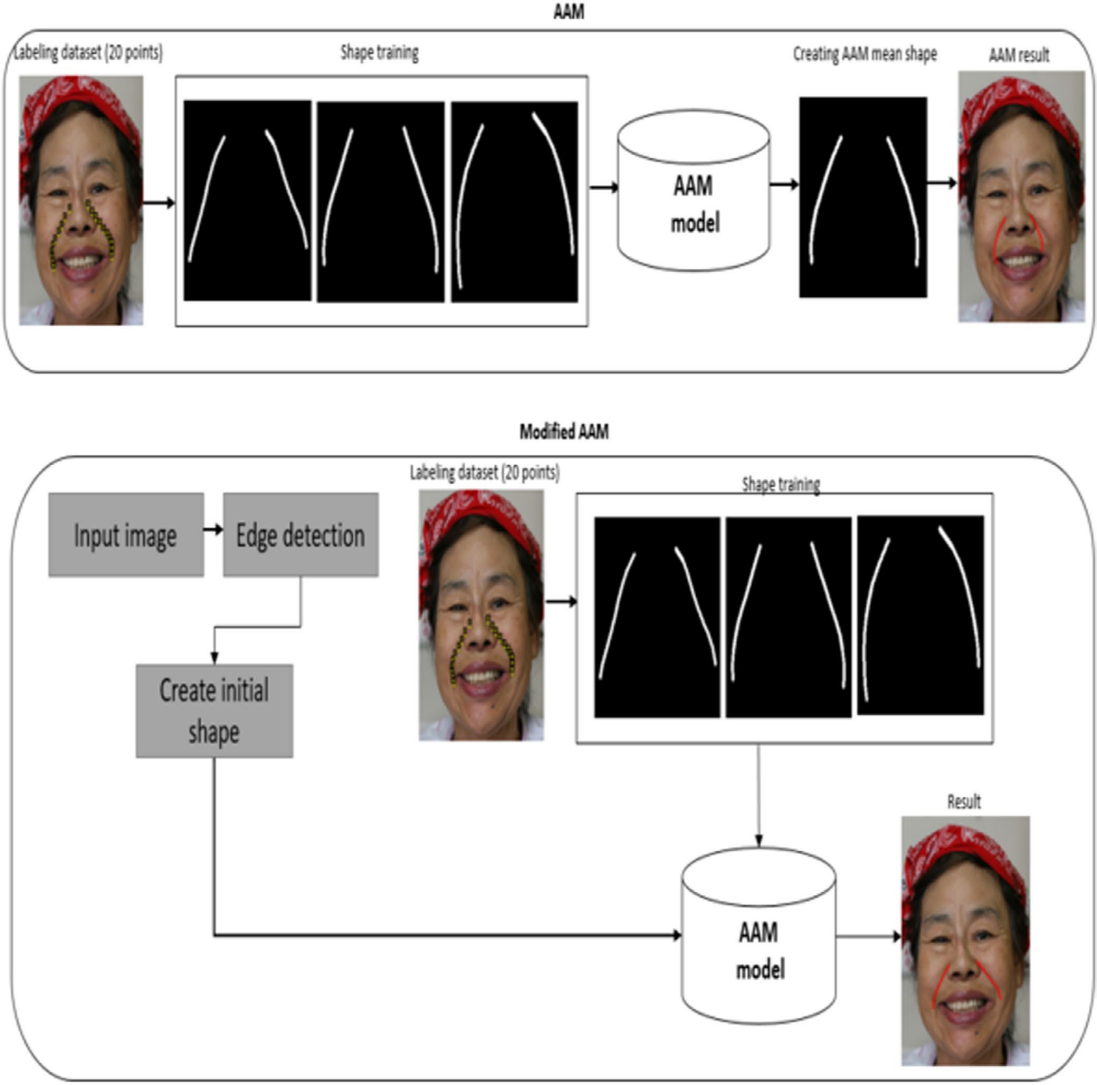
The framework illustrates wrinkle line detection differences between AAM and modified AAM [Colour figure can be viewed at wileyonlinelibrary.com]

AAM consists of shape and texture models^19^ Figure 2. Before creating the initial shape, the Procrustes transformation is applied to remove the shape differences of the training wrinkle structures using affine transformation. To retain the principal information of identity, pose, and expression, principal component analysis (PCA) is employed on the shape variations. For the new AAM model, the wrinkle detection structure $n={\left(x_{1},y_{1},\cdots ,x_{n},y_{n}\right)}^{T}$ represented as:

(3.11) $$s=msh_{0}+E_{p},p=E^{T}\left(n‐msh_{0}\right)$$

where *E* represents the Eigen vectors of shape, and *msh* represents new unique mean shape.

To apply the AAM algorithm for sets of points with unique initial shape under Procrustes transformation and reduce the error between input image and model using the relationship between the shape and texture model was applied:l.

(3.12) $$\min_{s}\frac{1}{2}\|vec‐W_{s}\|\begin{array}{c} 2\\ 2 \end{array}+\lambda \|s\|\begin{array}{c} 1 \end{array}$$

where *W* is a vectorization of wrinkle structure after Procrustes transformation, *s* is the spars coefficient corresponding to the wrinkle structure database, vec vec is the vectorization of the input shape, and λ is the regularization parameter 1e^−6^.

Algorithm 2. AAM Iteration with created unique mean shape.

**Table:** 

*Iteration = 1* ***while*** *Iteration < MaxIteration or no improvement is made to error E_0_* ***do*** *Sample image at (x,y)*→*g_image_* *Update model parameters* $p_{k}=Newp_{k‐1}‐\alpha \partial p$ *Update sample control points from (* ***Newx_model_*** *,* ***Newy_model_*** *) with similarity* *compositional pose correction*→*(x_k_, y_k_)* *Evaluate Error* $E_{k}=\partial g_{k}\partial g_{k}^{T}$ ***if*** *E_k_ < E_0_* ***then*** ***Accept model parameters,*** ${Newp}_{k}$ ***Accept control points (x,y) = (x_k_, y_k_)*** ***Update current error E_k_ = E_0_*** ***else*** *Try* α=1,α=0.5,α=0.25,α=0.125 ***end if*** *Iteration = Iteration +1* ***end while***

## 4 EXPERIMENTAL RESULTS

This research investigates the effectiveness of detecting nasolabial wrinkle lines based on AAM using a unique initial shape. The proposed algorithm successfully detected the wrinkle line in the nasolabial region, which compares with the performance of the proposed algorithm with state‐of‐the‐art algorithms. Figure 8 shows that the proposed method was much better in detecting the wrinkle line compared with other shape models.

### 4.1 Dataset and labeling

**FIGURE 3:**
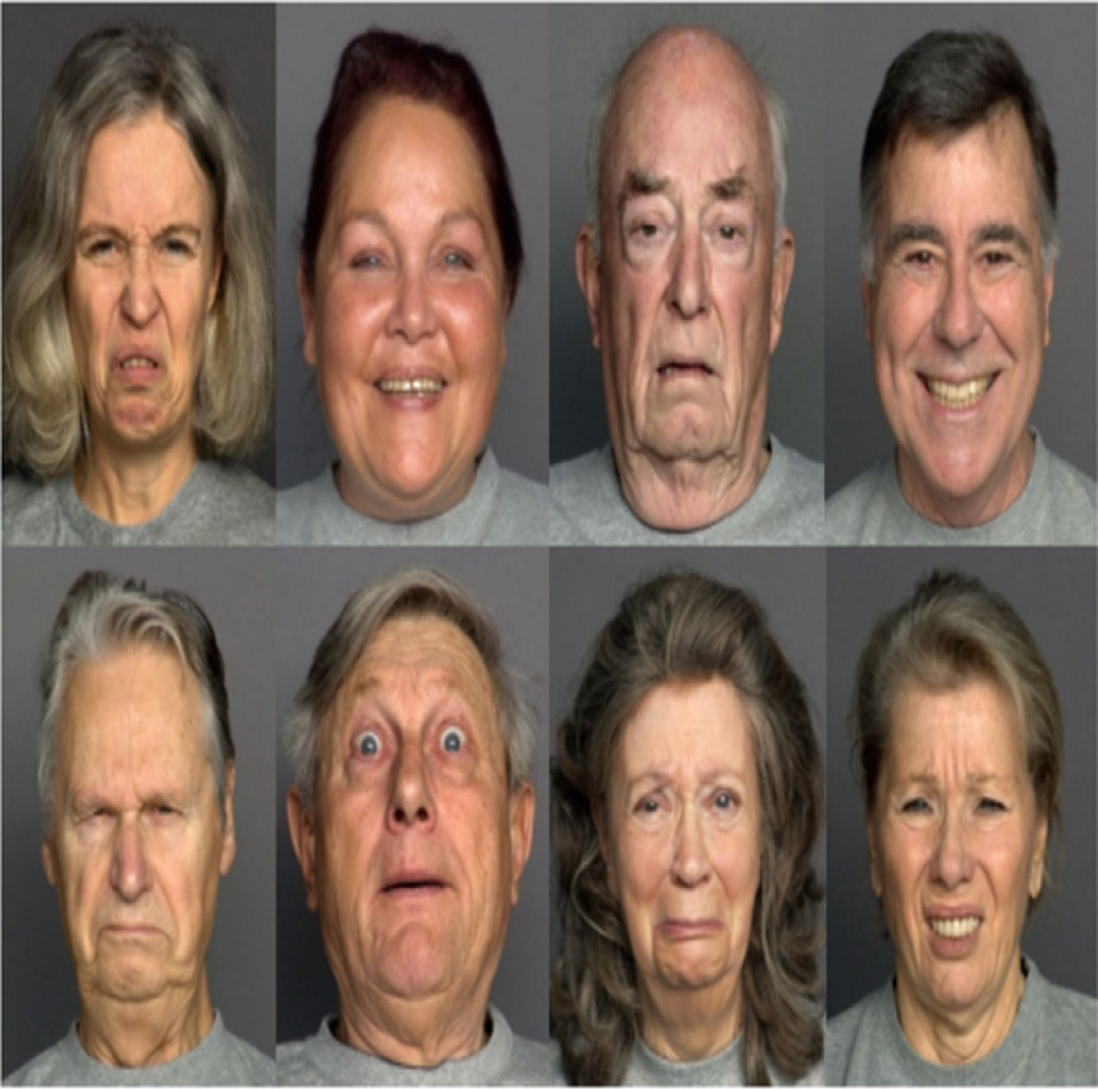
Illustration FACES dataset with different facial expressions [Colour figure can be viewed at wileyonlinelibrary.com]

**FIGURE 4:**
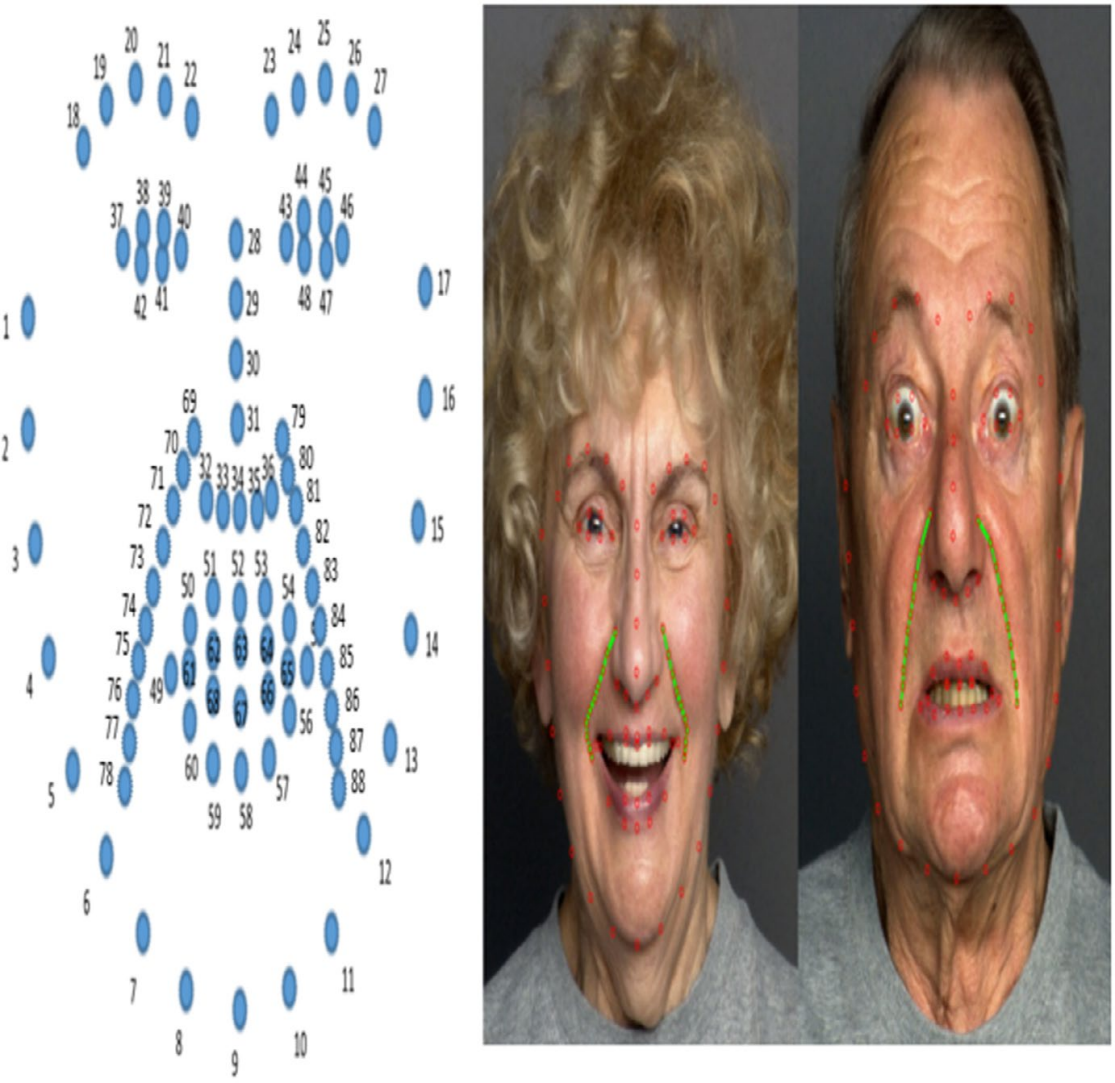
The 88 landmarks. 68 landmarks express face features illustrate as red points, and 20 landmarks illustrated as green line express nasolabial wrinkle positions [Colour figure can be viewed at wileyonlinelibrary.com]

The FACES dataset^20^ with different facial expressions images satisfies all requirements of this research Figure 3. Our research aims to get better results using a small number of images because within the dataset only 500 images were chosen for training and 100 images for testing. To locate the facial shape and key feature points for correspondence matching, 88 feature points including the nasolabial wrinkle line points are located by AAM^19^ and manually labeled based on Ref.,^21^, ^22^, ^23^, ^24^ Figure 4.

### 4.2 Face feature extracting and analyzing results

**FIGURE 5:**
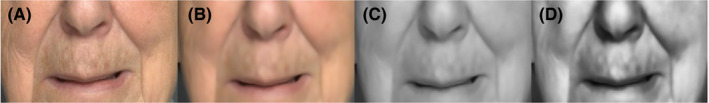
A, Original extracted part of the face, (B) Gaussian blurring, (C) the gray pattern of image, (D) automatic histogram equalization [Colour figure can be viewed at wileyonlinelibrary.com]

Our research is based on skin patterns that is why we were faced with problems such as noise and illumination. To overcome the skin pattern noise, illumination, shadow problems Gaussian blurring, and automatic histogram equalization methods were applied (Figure 5).

### 4.3 Localize wrinkle initial position result

**FIGURE 6:**
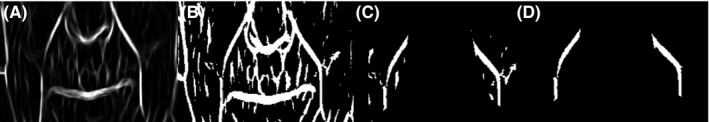
A, Wrinkle line position by Hessian matrix, (B) binary image, (C) region masked image, (D) discard some short and distorted lines less than LLT

The wrinkle initial position was detected by calculating Eigenvalues. The idea of the Hessian filter centers on the utilization of second‐order partial derivatives for edge detection. Eigenvalues of the Hessian filter were applied to extract principal directions into which the local second‐order structure of the image can be analyzed Figure 6.

Despite the fact that the Hessian filter benefits from the presence of the curve and valley extraction, a significant drawback is its omnidirectional nature. The vertical and horizontal discontinuities are detected as wrinkles; however, some of them are actually non‐wrinkles. To remain only wrinkle lines, the Equation (3.10) was implemented.

### 4.4 Wrinkle initial position result

**FIGURE 7 (A):**
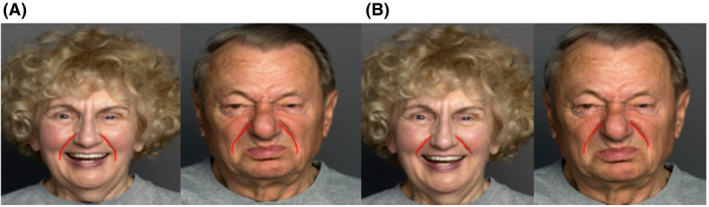
Initial shape token by AAM, (B) initial shape token using Hessian matrix and AAM [Colour figure can be viewed at wileyonlinelibrary.com]

In this research, for detecting the cheek wrinkle position was used for each input image unique initial shape based on edges of wrinkle line and AAM. Figure 7 illustrates two results: the first one (a) mean shape created by the appearance model and the second one (b) the mean shape created using second‐order derivatives. Since for every input image applied unique initial shape based on wrinkle shape characteristics, the curve lines detected more accurately than AAM.

### 4.5 Quantitative results

**FIGURE 8:**
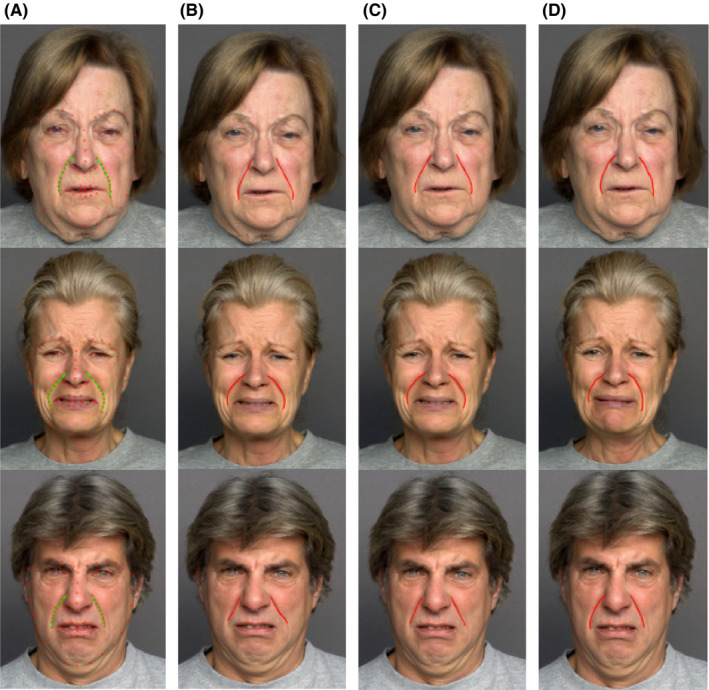
Nasolabial wrinkle line detection results. A, the ground truth, (B) ASM, (C) AAM, (D) the proposed method [Colour figure can be viewed at wileyonlinelibrary.com]

To compare the proposed method with state‐of‐the‐art algorithms, ASM‐based^15^, ^25^ and general AAM‐based^19^ edge‐based wrinkle detectors are implemented for comparison in which the same environment wrinkle initialization strategy Figure 8. In contrast, these two algorithms, the proposed method not only learns from the part deformation by constructing a wrinkle structure enclosing but also utilizes the texture information learned by the intrinsic function of AAM. The competitive ability of the proposed method can be proved by the observation that Jaccard Similarity Index (JSI)^24^ index values of the proposed method are better than those of the other two methods (Table 2). The proposed method not only estimates the positions of wrinkles but also created a new mean shape for every input that illustrated a better result on detecting wrinkle line position.

**Table 2:** Table 2 illustrates the results of calculation JSI between three algorithms

Method	ASM	AAM	The proposed method
JSI	0.85	0.92	0.98

The ASM‐based method^15^ detects cheek wrinkle line depending on other feature points geometrical features, and this method could not detect whether wrinkle lines have curves shape or sharp changings. The AAM method does not suitable to detect the different structures of shapes and does not sufficiently make use of local deformation around the wrinkle line. The competitiveness of the proposed algorithm can be proved by observation Figure 8 that is more accurate than those of the two methods. Moreover, the proposed algorithm estimated wrinkle line by modified AAM using the unique initial shape for each input image gave results more accurate than other methods, showing the effectiveness of this method.

In this paper, JSI was applied to calculate the error metric of detected wrinkle lines by calculating the similarity between manually labeled and detected wrinkle lines. An expansion of 20 points is applied to the assumed line when calculating the JSI. The Jaccard index:

(4.1) $$J\left(A,B\right)=\frac{\left|A\cap B\right|}{\left|A\cup B\right|}$$

where *A* the number of pixels on manually labeled wrinkle line, and *B* the number of pixels on detected wrinkle line. To validate the accuracy of the similarity between ground truth and the detected shape is defined as:

(4.2) $$\text{Accuracy}=\sum_{i=1}^{N}\begin{array}{c} J_{\mathrm{output}} \end{array},J_{\mathrm{output}}=\left\{\begin{array}{c} TrueifJ_{\mathrm{output}}>80\%\\ \mathrm{Falseelse} \end{array}\right)$$

where *N* is the total number of images 100, and *J* is output from Equation (4.1). If A ground truth and B output shape alignment, more than 80% decided as correct detection.

Table 2 illustrates the JSI statistics between ASM, AAM, and the proposed method. Overall, the accuracy of the proposed method got better results than the other two algorithms. Thus, the proposed algorithm illustrated that it was prone to localize wrinkle curves near the mouth tip and can be trained with the small dataset.

## 5 DISCUSSION AND CONCLUSION

The structure of wrinkles varies greatly in width, length, and pattern in different images, making it difficult to develop automatic wrinkle detection. Related works on the area of wrinkle line detection are mainly focused on detecting forehead and another part of the face based on filter and operators. Therefore, an efficient active appearance model has been proposed. This method is based on effective active appearance nasolabial wrinkle detection model, which apply the unique initial shape based on the wrinkle line structure.

In our study, was introduced the effectiveness of changing the structure of AAM and successfully applied in wrinkle line localizing. Although competitive results are achieved by the proposed wrinkle detection method, in the future, we planned to pay attention to skin texture information that can be used to achieve to create a wrinkle mapping model. In addition to wrinkle line structure, the effects of variation of color, face alignment, and illumination shall be studied in the future works.
